# PTBP1-mediated biogenesis of circATIC promotes progression and cisplatin resistance of bladder cancer

**DOI:** 10.7150/ijbs.96671

**Published:** 2024-06-24

**Authors:** Chenchen Huang, Yang Yang, Xiaofei Wang, Shuangchen Chen, Zhifu Liu, Zheng Li, Xingxing Tang, Qian Zhang

**Affiliations:** 1 Department of Urology, Peking University First Hospital, Beijing 100034, China.; 2 Institute of Urology, Peking University, Beijing 100034, China.; 3 Beijing Key Laboratory of Urogenital Diseases (Male), Molecular Diagnosis and Treatment Center, National Research Center for Genitourinary Oncology, Beijing 100034, China.; 4 Peking University Binhai Hospital, Tianjin, China.; 5 Peking University Shenzhen Hospital, China.

**Keywords:** bladder cancer, PTBP1, circATIC, RCC2, cisplatin resistance

## Abstract

**Background:** Cisplatin (DDP) based combination chemotherapy is a vital method for the treatment of bladder cancer (BLca). Chemoresistance easily occurs in the course of cisplatin chemotherapy, which is one of the important reasons for the unfavorable prognosis of BLca patients. Circular RNAs (circRNAs) are widely recognized for their role in the development and advancement of BLca. Nevertheless, the precise role of circRNAs in DDP resistance for BLca remains unclear.

**Methods:** To study the properties of circATIC, sanger sequencing, agarose gel electrophoresis and treatment with RNase R/Actinomycin D were utilized. RT-qPCR assay was utilized to assess the expression levels of circRNA, miRNA and mRNA in BLca tissues and cells. Functional experiments were conducted to assess the function of circATIC in BLca progression and chemosensitivity* in vitro*. Various techniques such as FISH, Dual-luciferase reporter assay, TRAP, RNA digestion assay, RIP and ChIRP assay were used to investigate the relationships between PTBP1, circATIC, miR-1247-5p and RCC2. Orthotopic bladder cancer model, xenograft subcutaneous tumor model and xenograft lung metastasis tumor model were performed to indicate the function and mechanism of circATIC in BLca progression and chemosensitivity *in vivo*.

**Results:** In our study, we observed that circATIC expression was significantly enhanced in BLca tissues and cells and DDP resistant cells. Patients with higher circATIC expression have larger tumor diameter, higher incidence of postoperative metastasis and lower overall survival rate. Further experiments showed that circATIC accelerated BLca cell growth and metastasis and induced DDP resistance. Mechanistically, alternative splicing enzyme PTBP1 mediated the synthesis of circATIC. circATIC could enhance RCC2 mRNA stability via sponging miR-1247-5p or constructing a circATIC/LIN28A/RCC2 RNA-protein ternary complex. Finally, circATIC promotes RCC2 expression to enhance Epithelial-Mesenchymal Transition (EMT) progression and activate JNK signal pathway, thus strengthening DDP resistance in BLca cells.

**Conclusion:** Our study demonstrated that circATIC promoted BLca progression and DDP resistance, and could serve as a potential target for BLca treatment.

## Background

Bladder cancer (BLca) is regarded as the most common malignancy of the urinary system, being disastrous to these patients 1. At present, the conventional treatment methods of BLca mainly include surgery, immunotherapy, chemotherapy and radiotherapy 2, 3. Surgery is recommended for early-stage patients instead of advanced BLca 4. Cisplatin is a standard first-line chemotherapy agent for patients with metastatic BLca, however, chemosensitivity severely limits its efficacy 5, 6. DDP resistance is inevitable in BLca treatment, causing recurrence, metastasis and poor prognosis in BLca 7, 8. The molecular mechanism of DDP resistance is still unclear, and elucidating the therapeutic target for DDP-resistance is of great clinical significance.

Circular RNAs, a type of conservative non-coding RNAs (ncRNA), are widely expressed in human tissues 9, 10. Recent studies found that circRNAs could participate in the regulation of tumor progression through various pathways 11-13. Accumulating evidence indicates that circRNAs are dysregulated in drug-resistant tissues, playing important roles in the resistance of chemotherapy drugs 14, 15. Mechanistically, circRNAs could sponge microRNAs and block microRNA-mediated mRNA silencing at the transcriptional levels 16, 17. For instance, circSTX6 promoted chemoresistance in BLca by sponging miR-515-3p 18. At the post-translational level, circRNAs could directly bind to proteins and regulate the progression of various cancers 19, 20. In BLca, circPTK2 promoted gemcitabine resistance by binding to PABPC1 21. CircRNAs can also play a role by regulating transcription and encoding peptides 22, 23. However, a large number of circRNAs that regulate BLca DDP-resistance are mysterious.

In this research, we identified that *hsa_circ_0058058*, termed as circATIC, was derived from PTBP1-mediated alternative splicing of ATIC. CircATIC expression was significantly increased in BLca tissues and cells and DDP-resistant cells. CircATIC expression are clearly associated with tumor size and postoperative metastasis, leading to a poor prognosis for these individuals. Tests conducted in cells and animals showed that circATIC enhanced the development and resistance to DDP treatment. Mechanistically, circATIC enhanced RCC2 expression in a ceRNA manner and increased stability of RCC2 mRNA by binding to LIN28A, thereby activating the JNK signaling pathway. Furthermore, circATIC promoted EMT-mediated DDP resistance in BLca cells and xenograft nude mice. Taken together, our study suggested that PTBP1-induced circATIC promoted BLca progression and DDP-resistance via RCC2/JNK signaling.

## Methods

### Clinical samples

The 52 pairs of clinical samples were obtained from the radical resection of bladder cancer and pathologically diagnosed as BLca in Peking University Shenzhen Hospital from 2009 to 2018. Table 1 displays the clinicopathological characteristics of the patients. The overall survival time of the patients within 5 years after surgery was followed up by telephone and outpatient follow-up. The Ethics Committee of Peking University Shenzhen Hospital granted approval for this study. Every patient participating in our study consented to the use of their clinical data and tissues for the experimental investigation and paper publication, and provided informed consent before samples were collected.

### Cell lines

BLca cell lines (5637, SW780, J82, T24) and normal bladder epithelial cell line (SV-HUC1) from humans were acquired from the American type culture collection Biological Resource Center in Manassas, Virginia. The cell lines referenced in our research were grown in full medium with DMEM (or RPMI-1640) supplemented with 10% fetal bovine serum and were placed in a cell incubator with 5% CO_2_ at 37 °C. DDP-resistant cell lines were incubated with the indicated medium with 1ug/ml DDP.

### Bioinformatic analysis

The data from TCGA database was download from the UCSC Xena website (https://xenabrowser.net). Circular RNA Interactome database (https://circinteractome.nia.nih.gov) was applied to forecast the potential miRNAs interacted with circATIC. miRDB (https://mirdb.org), Starbase (https://rnasysu.com/encori) and TargetScan (https://www.targetscan.org) databases were used to forecasted the specific genes bound with miR-1247-5p.

### RNA extraction and Real-time quantitative PCR

Total RNA was extracted from BLca samples collected from our hospital and BLca cells using Trizol reagent. To analyze RNA level at the subcellular level, we utilized the NE-PER Nuclear and Cytoplasmic Extraction Reagents (Thermo Scientific, USA) to partition total RNA into nucleus and cytoplasm. Reverse transcription kit from Toyobo were used for cDNA synthesis of mRNA and circRNA, while cDNA synthesis of miRNA was carried out using the All-in-One miRNA reverse transcription kit from GeneCopoeia, China. The RT-qPCR assay was performed using the SYBR Green Real-time PCR Master Mix from Toyobo, with the reaction conducted on the Roche LightCycler® 480II PCR machine in Basel, Switzerland. All primers included in our study were designed by the Primer-BLAST module in the NCBI database and presented in Supplementary Table S1.

### RNase R/Actinomycin D

To treat with RNase R, 1μg of RNA was mixed with 3U/μg RNase R (Epicenter, USA) and incubated at 37 °C for 1 hour. For Actinomycin D treatment, 5637 and SW780 were seeded in six-well plates and 5μg/ml actinomycin D was added when the cellular abundance reached 70-80%. Samples were collected at the specified time point and RNA expression was analyzed by RT-qPCR.

### Agarose gel electrophoresis

The cDNA was prepared with Emerald PCR Master Mix (TaKaRa, Japan), and 5μl was added to each hole of the 1% agarose gel. Electrophoresis was performed on 1X TAE for 20min. The images were captured with GBox Chemi-XL1.4 GeneSys system (SYNGENE, England).

### Fluorescence *in situ* hybridization (FISH)

Glass slides were used to seed cells in a 24-well plate. Upon reaching a cellular abundance of 70%, the cells were treated in accordance with the guidelines provided by the Fluorescence *in situ* hybridization kit from RiboBio, China. The circATIC probe (obtained from RiboBio) was used for incubation during the period. Images were captured by laser confocal microscopy (Leica, Germany) after sealing the slides.

### Cell transfection

Plasmid transfection was carried out after cells were placed in six-well plates and reached a density of 50-70%. Cells were starved for 1-2 hours in advance. The transfection mixture was created using the plasmids (obtained from GenePharma, Suzhou, China) and Lipofectamine 3000 transfection agent (Invitrogen, USA) following the provided guidelines. Following the creation of a DNA-liposome compound, the transfection solution was gently added to the cells in droplets. After 48 hours of transfection, relevant experimental analysis can be performed. For stable transfection, the lentivirus-packaged plasmids (purchased from GenePharma) were added to BLca cells. After 1-2 week of drug screening, stably transfected cell lines were obtained, which could be used for the subsequent experiments. The plasmid sequences used in this research can be found in Supplementary Table S2.

### Cell viability, CCK-8 and Colony formation assay

Transfected cells were plated at a density of 1000 cells per well in 96-well plates for cell viability and CCK-8 testing. CCK-8 reagent (KeyGEN BioTECH, China) was added at 0, 24, 48, and 72 hours, respectively. After incubating for 1 hour, the microplate reader from Bio-Rad in the USA was used to measure absorbance. Transfected cells were plated in 6-well dishes at a density of 1000 to 2000 cells per well for the colony formation test. After 2 weeks, we stained the cell with 0.1% crystal violet. The absorbance of the staining solution was measured at 590nm using the microplate reader.

### 5-Ethynyl-20-deoxyuridine (EdU) assay

Cell proliferation was detected by EdU proliferation assay kit (RiboBio, China). Cells that underwent transfection were placed onto glass slides within 12-well culture dishes. Once the cells reached a 40% abundance, a low concentration of EDU solution was introduced and left to incubate for 24 hours. Following the manufacturer's instructions, the cells were first treated with 4% paraformaldehyde for fixation, then permeabilized with Triton, and finally stained with Cy3 and DAPI. Photographs were then taken with a fluorescence microscope (Olympus, Japan).

### Cell migration and invasion assays

For wound-healing assays, transfected cells were seeded in 6-well plates and grown to 100% density. Photographs were taken after using a pipette tip to make a cross-shaped wound in the cell layer. After 24~48h of incubation with the medium containing 2% serum, photographs were taken again. The difference in cell migration distance was analyzed. In transwell migration experiments, 2000 cells that had been transfected were placed in the upper chamber with 100μl of medium without serum, while the lower chamber received complete medium. Following a 48 to 72-hour incubation period, cells that had entered the compartments were treated with a 0.1% crystal violet solution for 30 minutes and then photographed. In preparation for the transwell invasion assay, the chamber was pre-incubated at 37 °C for 1 hour after adding a diluted solution of Matrigel from Corning, USA. The following experimental method closely resembled the transwell migration assay. The absorbance of the crystal violet solution was measured at 590nm using the microplate reader.

### Tagged RNA Affinity Purification (TRAP) assay

Cells were transfected with both the GST-MS2 fusion expression vector and the circATIC-MS2 stem-loop tandem repeat vector (created by BersinBio, China) to produce complexes of GST-MS2~MS2-circATIC-binding protein or RNA. Samples were washed and purified according to the instructions of the TRAP kit (BersinBio 5106). CircATIC-bound RNA or protein was obtained, and the products were further analyzed using RT-qPCR or Western blotting assay. The circRNA independent expression vector was co-transfected with GST-MS2 as a negative control, since it could not form a complex with GST-MS2.

### Dual luciferase reporter gene assay

According to the binding site between circATIC and miR-1247-5p, dual luciferase reporter gene vectors containing circATIC wild type sequence (WT circATIC) and mutant sequence (Mut circATIC) were synthesized. According to the binding site between RCC2 and miR-1247-5p, dual luciferase reporter gene vectors containing RCC2 wild type sequence (WT RCC2) and mutant sequence (Mut RCC2) were synthesized. After co-transfection of the reporter plasmid with NC mimic or miR-1247-5p mimic, dual luciferase reporter reagent (Promega, WI, USA) was added. Luciferase activity in the transfected cells was quantified using a microplate reader, measuring two different activities. ALL Dual luciferase reporter vector were purchased from GenePharma. The sequences of the above vectors were listed in Supplementary Table S2.

### Chromatin Isolation by RNA Purification (ChIRP) assay

Based on the specific region of PTBP1 binding to ATIC pre-mRNA, we purchased the 5'-biotin-labeled ChIRP probe, Biotin-preATIC, from RiboBio. ChIRP assays were carried out following the instructions provided in the ChIRP kit manual (BersinBio 5104-2). The final products obtained were further analyzed by western blotting assay. A probe designed according to the lacZ gene was used as a negative control. The probe sequence was listed in Supplementary Table S2.

### RNA immunoprecipitation (RIP)

Cells were processed in accordance with the guidelines provided in the RIP kit (BersinBio 5101). Magnetic beads coated with protein A/G were treated with an anti-Lin28A antibody from Proteintech, USA, or a negative control IgG antibody. Subsequently, antibodies were incubated with BLca cells. The coprecipitated RNA was then purified and enriched by qRT-PCR assay. The primer sequences can be found in Supplementary Table S1.

### Western blotting assay

Total proteins were extracted from the cells using RIPA lysis buffer and protease inhibitors (Beyotime, China). Proteins were separated by SDS-PAGE gel electrophoresis at 100V for 90min. Following electroporation at 330 mA for 70 minutes, the proteins were then moved onto PVDF membranes with a pore size of 0.45 μm. After blocking with 5% milk, membranes were incubated overnight at 4 °C with primary antibodies (as detailed in Supplementary Table S3), then treated with secondary antibodies (#7074). Exposure was carried out using a chemiluminescence kit from Millipore in Billerica, USA, and images were captured with the BioSpectrum 600 imaging system from UVP in CA, USA.

### Immunohistochemistry (IHC) and hematoxylin-eosin (HE) staining

Immunohistochemistry kit (ZSGB-BIO, China) was used to assist IHC experiments. After the tissues were embedded in paraffin, they were sectioned. The paraffin sections were baked in an oven at 70 °C for 1 hour. The sections were deparaffinized and antigen retrieval. An inhibitor of peroxidase was applied to the sections and left to incubate for 20 minutes at ambient temperature. Sections were treated with goat serum for 30 minutes to block, then left overnight at 4 °C with primary antibodies (refer to Supplementary Table S3) for incubation. Following that, the segments were exposed to secondary antibodies (#7074) for 1 hour at 4 degrees Celsius. With microscope monitoring, DAB solution was dropped to select the appropriate color development time. After hematoxylin staining, the cells were soaked under water for 30min, and hydrated. Finally, the sections were sealed with resin and allowed to dry overnight. Photos were taken using a microscope. For HE staining, there were no antibodies and only hematoxylin and eosin dyes were used for staining to facilitate visualization of the tissue structure.

### Xenograft model

Animal experiments were approved by the Animal Ethics Committee of Peking University First Hospital. For the xenograft subcutaneous tumor model, SW780 cells were infected with lentiviruses (shNC or shcirc-1) to obtain two groups of stable transfected cells. Two groups were formed with fourteen BALB/c nude mice that were 4 weeks old, selected at random. 1x10^7^ infected cells were injected subcutaneously into each nude mouse on their back. Tumor volume was measured weekly, and subcutaneous tumors were removed after 5 weeks for weighing and IHC experiments. For orthotopic bladder cancer model, 5×10^4^ cells were seeded in the bladder for each 4-week-old C57B6J mouse. After 1 month, the luminescence of the tumor was detected and the mice were sacrificed for tumor collection. Twelve 4-week-old BALB/c nude mice were randomly assigned to either the NC group (shNC) or the shcircATIC (shcirc-1) group for the xenograft lung metastasis tumor model. Each nude mouse was injected with 2×10^6^ infected SW780 cells via the tail vein. Following a period of 1 to 2 months, the nude mice were euthanized, and their lung tissues were harvested for nodule counting and HE staining. For DDP-resistant model, each mouse was injected with PBS or DDP via the tail vein after models were constructed. The nodules were collected for primary cell culture, and were repeatedly injected subcutaneously into mice. The fourth passage of cells was collected and named DDPR4 for subsequent studies.

### Statistical analysis

Data from experiments were analyzed using statistical software including GraphPad Prism and SPSS (IBM, USA). Clinical sample analysis utilized paired sample t-test and Chi-square test. Overall survival time was assessed using the Kaplan-Meier assessment and log-rank analysis. Groups were compared using either a student's t-test or ANOVA. Linear regression analysis was used for correlation analysis. A P value less than 0.05 was deemed to be statistically significant and denoted by *, while a P value less than 0.01 was denoted by **.

## Results

### Expression and clinical characterization of circATIC in BLca

**Figure 1:**
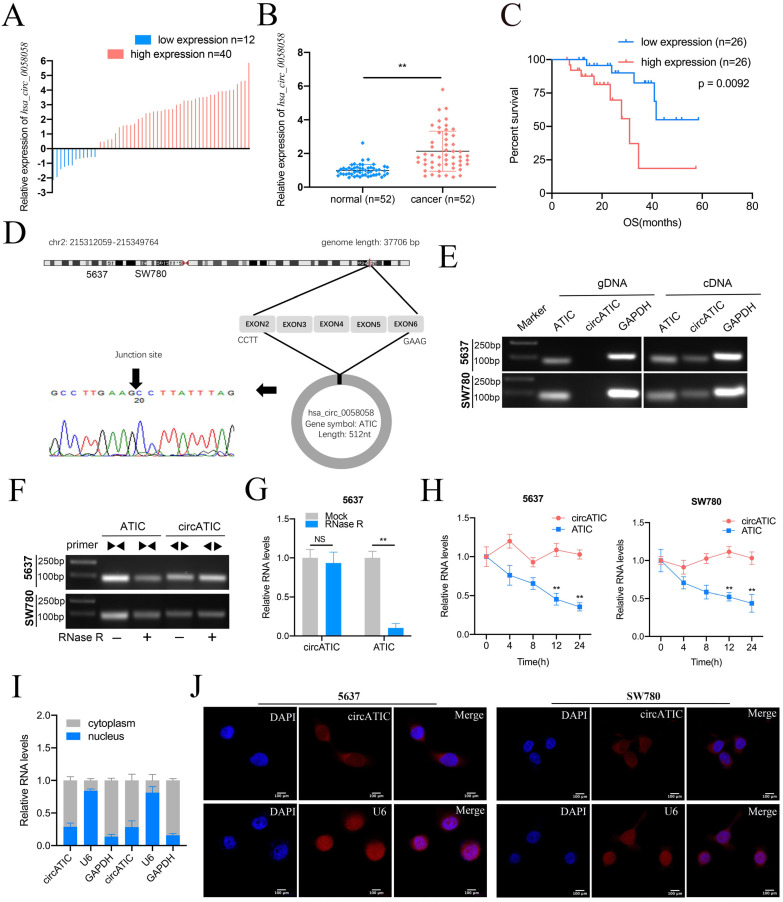
The features of circATIC in BLca. A. The expression level of circATIC in 52 BLca tissues. B. Relative expression level of circATIC in 52 BLca tissues and paired normal tissues. C. The OS time in BLca patients with different expression level of circATIC. D. Sanger sequencing verified the sequence of circATIC. E. Agarose gel electrophoresis assay verified the existence of circATIC in BLca cancer cells. F. Agarose gel electrophoresis assay showed that circATIC was resistant to RNase R digestion. G. RT-qPCR assay showed that circATIC was resistant to RNase R digestion. H. The expression level of circATIC and ATIC mRNA in the condition of amphotericin treatment. I-J. Nuclear and cytoplasmic fractionations and FISH assay showed that circATIC was dominantly located in cytoplasm. U6 and GAPDH were used as parallel controls.

To evaluate hsa_circ_0058058 expression level in BLca tissues and normal tissues, we performed qRT-PCR assay and discovered that hsa_circ_0058058 expression was dramatically increased in 76.9% (40 of 52) cancer tissues (Fig. 1A and B). Elevated level of hsa_circ_0058058 is associated with larger tumor size and appearance of postoperative metastasis (Table 1). In addition, high hsa_circ_0058058 expression in BLca tissues was negatively correlated with a lower 5-year overall survival rate (Fig. 1C). Hsa_circ_0058058, termed as circATIC, was derived from the exon 2 to 6 of ATIC mRNA and created a 512 nt circular transcript (Fig. 1D). circATIC could be detected in cDNA of BLca cells instead of gDNA (Fig. 1E), indicating that circATIC is a circular transcript. RNase R and Actinomycin D digestion assay demonstrated that circATIC was able to resist the digestion. However, ATIC mRNA failed to resist the digestion of RNase R (Fig. 1F-H). Finally, nuclear mass separation and RNA-FISH assay demonstrated that circATIC was mainly located in the cytoplasm (Fig. 1I and J), suggesting that circATIC may serve as a miRNA sponge.

### PTBP1-induced circATIC facilitated the progression of BLca *in vitro* and *in vivo*

**Figure 2:**
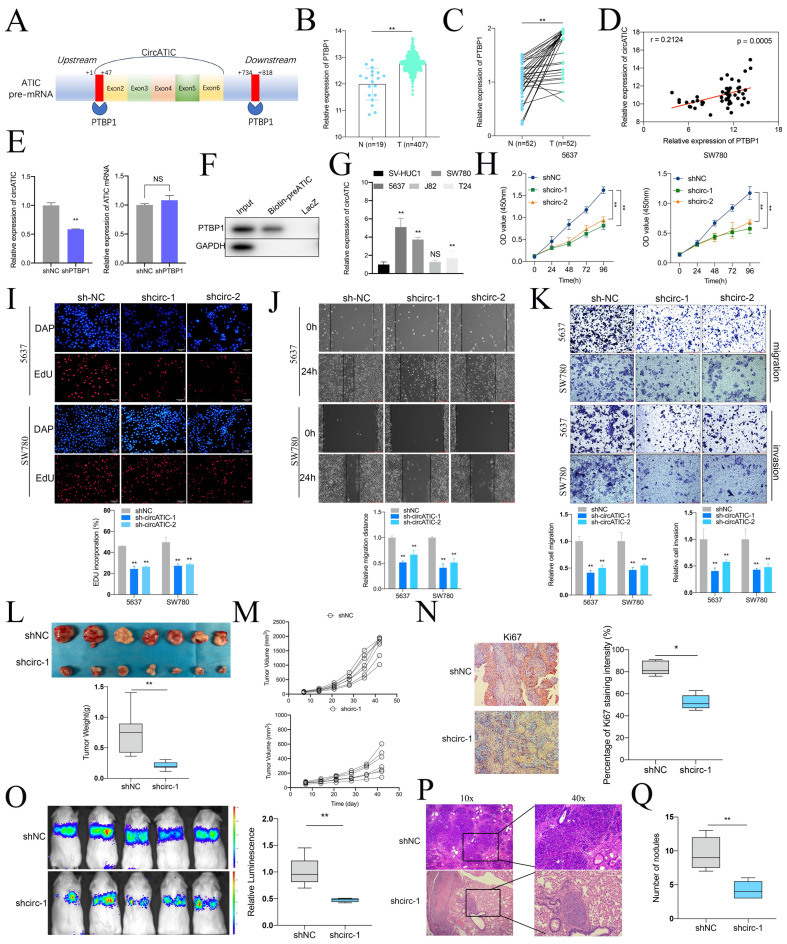
CircATIC induced by PTBP1 promoted the progression of BLca *in vitro* and *in vivo*. A. Two binding sites between PTBP1 and ATIC pre-mRNA were predicted by CircInteractome database. B. The expression level of PTBP1 in BLca tissues compared to normal tissues from TCGA database. C. The expression level of PTBP1 in BLca tissues compared to normal tissues from our hospital. D. The correlation between PTBP1 expression and circATIC expression in BLca tissues from our hospital. E. The expression level of circATIC and ATIC mRNA upon PTBP1 knockdown. F. ChIRP assay confirmed that PTBP1 was enriched on circATIC pre-mRNA. G. The expression level of circATIC in BLca cell lines and bladder urothelial normal cell line. H. CCK-8 assay showed the growth curve of of BLca cells upon PTBP1 knockdown. I. EdU assay showed the proliferation rate of BLca cells upon PTBP1 knockdown. J-K. Wound-healing assay and transwell assay showed that the migrative and invasive activity of BLca cells upon PTBP1 knockdown. L. The weight of subcutaneous xenograft tumors was measured. M. The volume of subcutaneous xenograft tumors was measured. N. IHC staining of Ki67 in the tumors from shNC or shcirc-1 group. O. The luminescence of lung metastatic nodules in mice from shNC or shcirc-1 group were tested. P and Q. The number of lung metastatic nodules from shNC or shcirc-1 group.

Alternative splicing is an important pathway for circRNAs synthesis, and splicing enzymes promote or inhibit the synthesis of circRNAs by binding to the intronic sequences flanking the exons. According to the CircInteractome database, PTBP1 bound to the flanking intron region of pre-ATIC (Fig. 2A, Fig. S1A). Alternative splicing enzyme PTBP1, also known as PTB or hnRNP, was reported to be a modulator in the biogenesis of circRNAs 24-26. Compared to normal tissues, PTBP1 mRNA expression was obviously increased in BLca tissues from TCGA database (Fig. 2B) and our BLca samples (Fig. 2C). Besides, PTBP1 mRNA expression was positively correlated to circATIC expression (Fig. 2D). Knockdown of PTBP1 significantly inhibited the expression of circATIC instead of ATIC mRNA (Fig. 2E). To clarify the mechanism by which PTBP1 promotes circATIC synthesis, we performed ChIRP assay. The 5 '-biotin labeled pre-mRNA of ATIC, containing the PTBP1 binding sequence, significantly enriched PTBP1 protein compared to LacZ (Fig. 2F).

Next, qRT-PCR showed that 5637 and SW780 cells own the highest circATIC expression (Fig. 2G). Two short-harpin RNAs (shRNAs) targeting circATIC (shcirc-1 and shcirc-2) were applied to impair circATIC expression (Fig. S1B). The CCK-8 and EdU experiment indicated that reducing circATIC expression notably reduced the growth rate of BLca cells (Fig. 2H and I). Decreased circATIC expression was shown to inhibit BLca cell migration and invasion, as demonstrated by wound-healing and transwell assay results (Fig. 2J and K). Furthermore, xenograft models were established to investigate the impact of circATIC on the growth and metastasis of BLca *in vivo*, including subcutaneous and lung metastasis tumors. Tumors from the circATIC knockdown group had noticeably lower weight and volume compared to the negative control group (Fig. 2L and M). IHC assay suggested that suppressed circATIC expression restrained Ki67 expression (Fig. 2N). Xenograft lung metastasis tumor model showed that knockdown of circATIC effectively reduced the metastasis of BLca (Fig. 2O). According to the HE staining, the number of lung nodules in circATIC knockdown group mice was less than that in the negative control group (Fig. 2P and Q). The findings validated that circATIC induced by PTBP1 promoted the proliferation and spread of BLca cells both in laboratory settings and in living organisms.

### circATIC acts as a miR-1247-5p sponge

**Figure 3:**
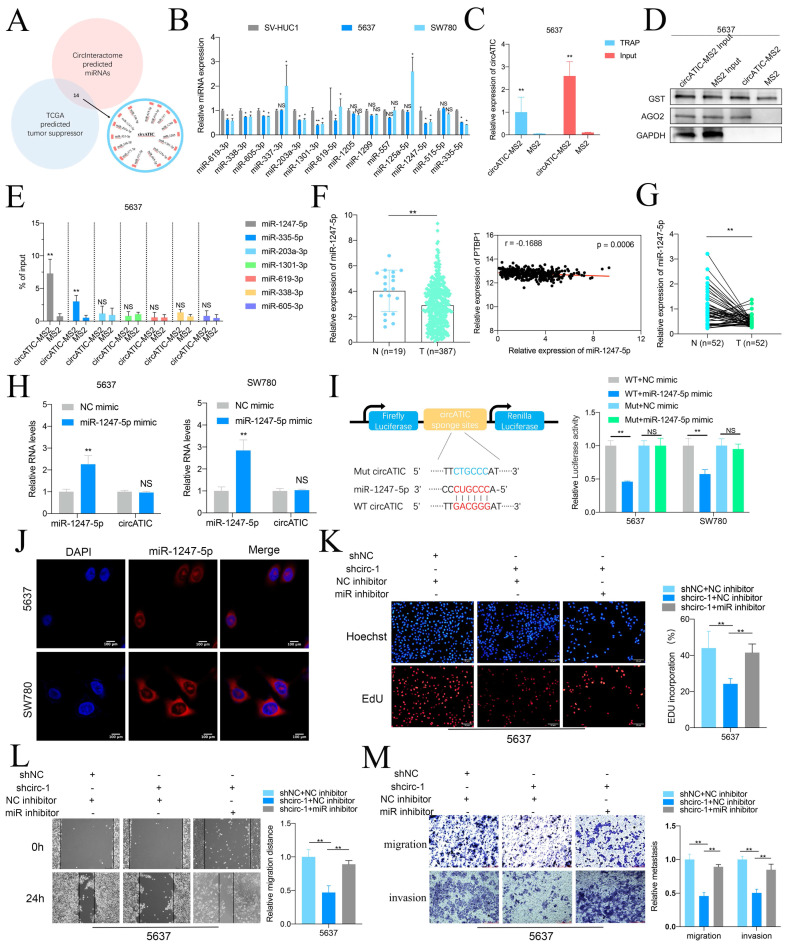
CircATIC sponged miR-1247-5p. A. TCGA database and CircInteractome database predicted 14 miRNAs candidates potentially binding to circATIC. B. RT-qPCR assessed the expression of miRNA candidates in BLca cell lines. C. TRAP assay confirmed that we obtained circATIC-MS2 complex comparing to the negative control (MS2). D. Western blotting showed that AGO2 was enriched on circATIC-MS2 complex. E. RT-qPCR showed that miR-1247-5p was significantly enriched on the circATIC-MS2 complex. F. miR-1247-5p expression in BLca tissues and normal tissues from TCGA database and the correlation between miR-1247-5p expression and PTBP1 expression in BLca tissues from TCGA database. G. miR-1247-5p expression in BLca tissues compared to normal tissues from our hospital. H. miR-1247-5p expression in BLca cells upon overexpression of miR-1247-5p. I. Luciferase activity showed that circATIC specifically sponged miR-1247-5p via the core binding sequence. J. FISH assay showed that miR-1247-5p was dominantly located in cytoplasm. K. EdU assay showed that decreased miR-1247-5p (miR inhibitor) rescued the proliferation inhibited by circATIC knockdown. L-M. Wound-healing assay and transwell assay showed that the inhibition of migration and invasion of BLca cells induced by circATIC knockdown was rescued by silencing of miR-1247-5p (miR inhibitor).

As circATIC is predominantly located in cytoplasm and predicted to bind AGO2 (Fig.S2A), circATIC may serve as a miRNA sponge. The Circular RNA Interactome database was utilized to predict the miRNAs that may be associated with circATIC (Fig. S2B). Fourteen potential miRNAs that could bind were selected, which include miR-1247-5p, miR-203a-3p, miR-335-5p, miR-338-3p, miR-377-3p, miR-515-5p, miR-605-3p, miR-125a-5p, miR-1301-3p, miR-1205, miR-1299, miR-557, miR-619-3p, miR-619-5p, and miR-338-3p (Fig. 3A). Oncogenic circRNAs absorb tumor suppressor miRNAs to exert its biological function. Fourteen miRNAs were assessed in BLca cells and SV-HUC1, resulting in the identification of seven miRNAs: miR-1247-5p, miR-335-5p, miR-203a-3p, miR-1301-3p, miR-619-3p, miR-338-3p, and miR-605-3p (Fig. 3B). The TRAP study showed a notable increase of miR-1247-5p on circATIC, attracting the RNA-induced silencing complex (RISC) during the pull-down process (Fig. 3C-E). Consistently, miR-1247-5p is significantly decreased in BLca tissues, and miR-1247-5p expression is negatively associated with PTBP1 expression according to bioinformatics (Fig. 3F, Fig. S2C). Besides, miR-1247-5p expression is also decreased in BLca tissues from our cohort (Fig. 3G). Overexpression of miR-1247-5p could not affect circATIC expression (Fig. 3H). To detect the core binding sequence between circATIC and miR-1247-5p (Fig. S2D), we constructed dua-luciferase reporter assay (Fig. 3I). Forced miR-1247-5p expression significantly inhibited the luciferase activity of WT circATIC vector instead of Mut circATIC vector, suggesting that miR-1247-5p binds to the specific sequence of circATIC (Fig. 3I). Overexpression of miR-1247-5p effectively suppressed luciferase activity in the wild-type circATIC vector rather than the mutant circATIC vector, indicating a direct binding of miR-1247-5p to a specific sequence within circATIC (Fig. 3I). The RNA-FISH test verified that miR-1247-5p was situated in the cytoplasm, similar to circATIC (Fig. 3J).

Finally, rescue assay between circATIC and miR-1247-5p was performed. The EdU test showed that blocking miR-1247-5p reversed the circATIC reduction's ability to inhibit cell proliferation (Fig. 3K). Decreased expression of miR-1247-5p was shown to reverse the inhibitory impact of circATIC reduction on cell migration and invasion, as evidenced by wound healing and transwell assays (Fig. 3L and M). These results suggested that circATIC exerts its biological function via absorbing miR-1247-5p.

### miR-1247-5p suppresses BLca progression by targeting RCC2

**Figure 4:**
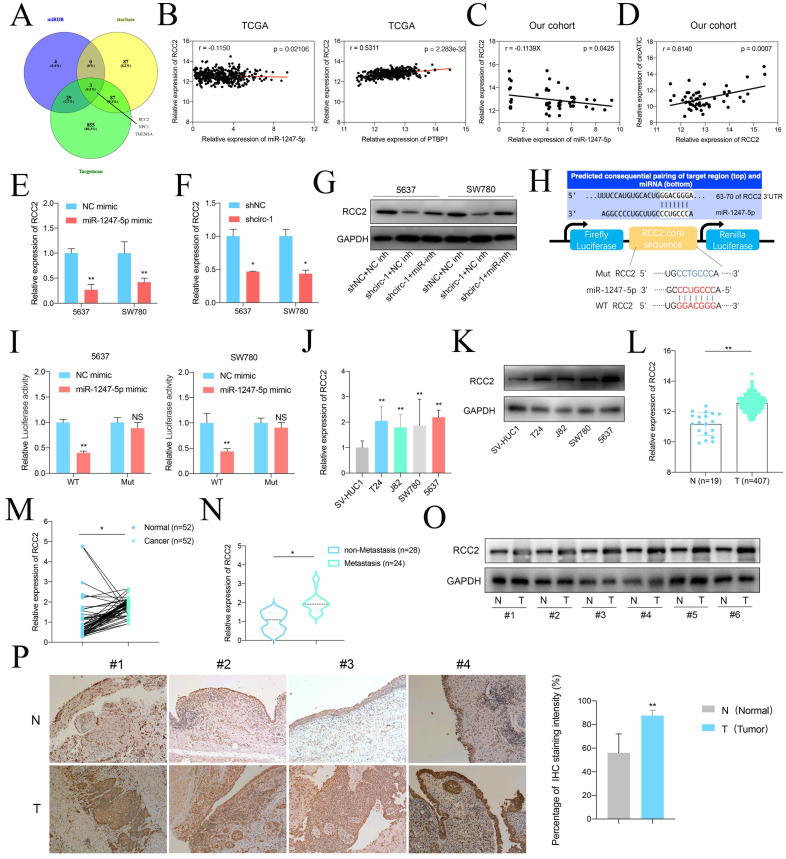
miR-1247-5p directly targeted RCC2. A. miRDB, starbase and Targetscan predicted the potential target genes of miR-1247-5p. B. The correlation between miR-1247-5p expression and RCC2 mRNA expression in BLca tissues from TCGA database. The correlation between PTBP1 mRNA expression and RCC2 mRNA expression in BLca tissues from TCGA database. C. The correlation between miR-1247-5p expression and RCC2 mRNA expression in BLca tissues from our hospital. D. The correlation between circATIC expression and RCC2 mRNA expression in BLca tissues from our hospital. E. Forced expression of miR-1247-5p induced down-regulation of RCC2 mRNA. F. CircATIC inhibition induced down-regulation of RCC2. G. Knockdown of circATIC inhibited RCC2 protein, while knockdown of miR-1247-5p (miR-inh) reversed this effect. H. Schematic diagram of wide-type dual-luciferase reporter vector (WT RCC2) and mutant (Mut RCC2) regarding the indicated site. I. Dual-luciferase reporter assay showed that miR-1247-5p bounded to the specific binding sequence on RCC2 3'UTR. J-K. RCC2 mRNA expression level in BLca cell lines and SV-HUC1 cell. L. RCC2 mRNA expression in BLca tissues from TCGA database. M. RCC2 mRNA expression in BLca tissues from our hospital. N. RCC2 mRNA expression in BLca tissues from our hospital. O. Western blotting assay showed the RCC2 protein level in BLca tissues (T) and paired normal tissues (N). P. IHC staining of RCC2 in BLca tissues and paired normal tissues.

Based on the relationship between circATIC and miR-1247-5p, we investigate the biological role of miR-1247-5p. Several research studies have shown that miR-1247-5p functions as a cancer suppressor in different types of cancers, including breast cancer, hepatocellular carcinoma and astroglioma 27-29. To forecast the potential genes regulated by miR-1247-5p, we performed bioinformatics analysis through utilizing miRDB, Starbase and TargetScan databases and discovered three genes interacted with miR-1247-5p, including RCC2, NPC1, and TMEM8A (Fig. 4A). Contrary to NPC1 and TMEM8A, a thorough examination of transcriptional data from the TCGA database revealed that RCC2 expression had a negative correlation with miR-1247-5p expression and a positive correlation with PTBP1 expression (Fig. 4B, Fig. S3A-D). Extensive analysis of transcription within our group revealed a negative correlation between miR-1247-5p levels and RCC2 expression (Fig. 4C) and circATIC expression was positively associated with RCC2 expression (Fig. 4D). Overexpression of miR-1247-5p or suppression of circATIC could inhibit RCC2 mRNA expression (Fig. 4E and F). In addition, suppression of miR-1247-5p could reverse the inhibitory effect of circATIC suppression on RCC2 protein suppression (Fig. 4G). In order to determine if RCC2 could be targeted by miR-1247-5p, a luciferase reporter plasmid was created using the WT or Mut sequence of RCC2 3'UTR with the miR-1247-5p binding site (Fig. 4H). Luciferase reporter assay demonstrated that miR-1247-5p directly bound to the 3'UTR of RCC2 (Fig. 4I).

Next, we observed that RCC2 mRNA and protein expression were increased in BLca cells (Fig. 4J and K). RCC2 mRNA expression was not only dramatically increased in tumor tissues from TCGA database and our samples (Fig. 4L and M) but also significantly upregulated in BLca patients who have developed metastasis after surgery. Western blot and IHC assay demonstrated that RCC2 protein expression was dramatically increased in BLca tissues (Fig. 4O and P). These results suggest that miR-1247-5p promotes BLca progression by targeting RCC2.

### RCC2 overexpression recovers the inhibitory effects of circATIC reduction on cell growth and metastasis *in vitro* and *in vivo*

**Figure 5:**
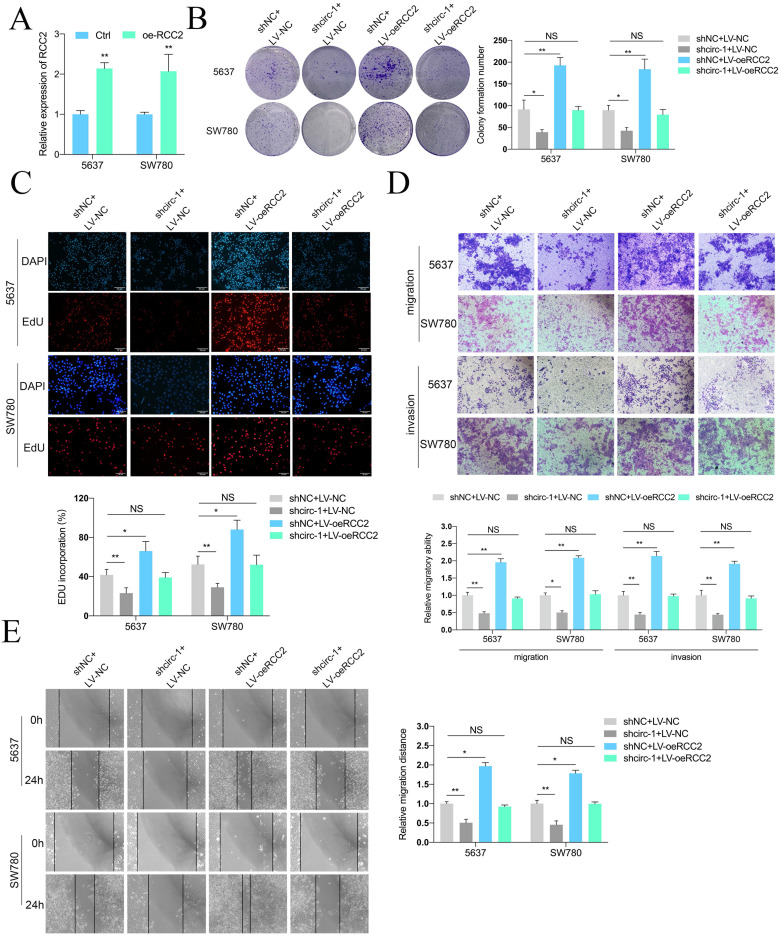
Forced RCC2 expression rescued the inhibition of BLca progression induced by circATIC knockdown *in vitro*. A. RT-qPCR confirmed the efficiency of RCC2 over-expression (oe-RCC2). B-C. Colony formation assay and EDU assay showed that over-expression of RCC2 (LV-oeRCC2) reversed the proliferative inhibition induced by circATIC knockdown. D. Wound-healing assay showed that over-expression of RCC2 reversed the migrative inhibition induced by circATIC knockdown. E. Transwell assay showed that over-expression of RCC2 reversed the migrative and invasive inhibition induced by circATIC knockdown.

To investigate whether circATIC exhibits its role through via enhancing RCC2 expression, a rescue assay between circATIC and RCC2 was carried out. Colony formation and Edu assay showed that upregulation of RCC2 reversed the inhibition of cell proliferation caused by circATIC silencing (Fig. 5A-C). The transwell migration and invasion assay showed that increased RCC2 reversed the inhibition of cell migration and invasion caused by circATIC silencing (Fig. 5D). Wound healing assay also confirmed that enhanced RCC2 reversed the circATIC silencing-induced cell migration inhibition (Fig. 5E).

**Figure 6:**
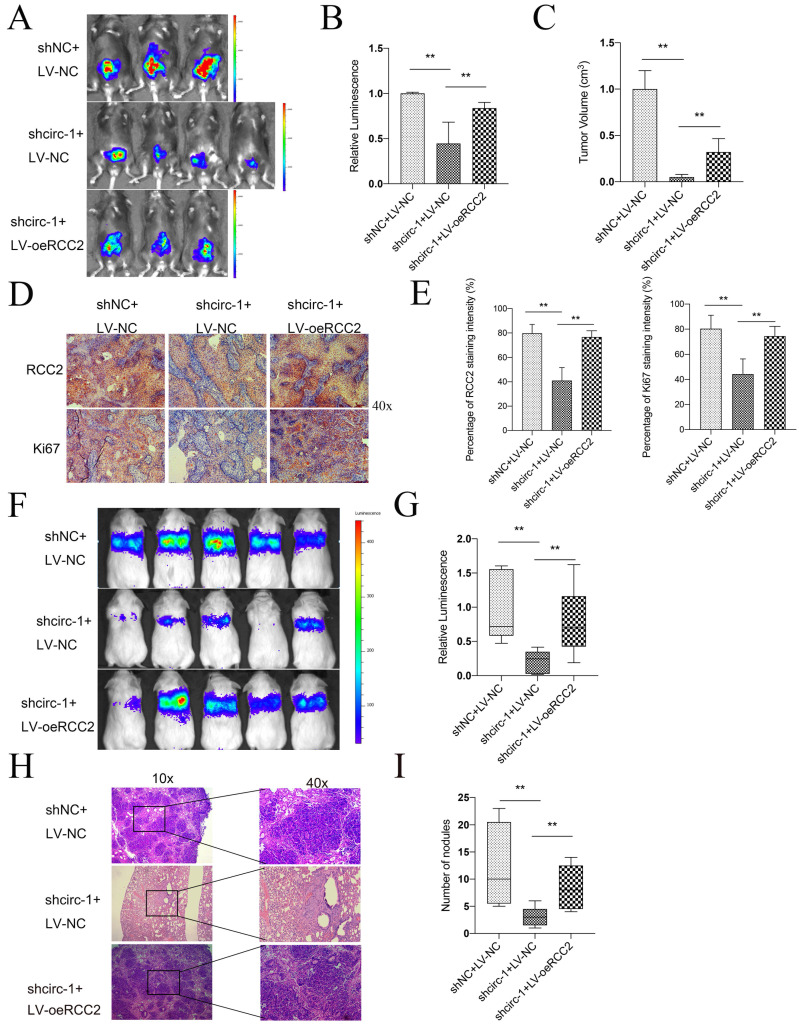
Forced expression of RCC2 rescued the inhibition of BLca progression induced by circATIC knockdown *in vivo*. A. The luminescence of tumor within the Orthotopic bladder cancer model were measured. B. Statistical plot of luminescence in each group. C. The volume of harvested bladder tumors in each group. D. IHC staining of RCC2 and Ki67 in harvested bladder tumors. E. Statistical plot of IHC staining intensity of RCC2 and Ki67. F-G. The luminescence of lung metastatic nodules in mice were tested after one month. H. Comparison of lung metastatic nodules by HE staining. I. The number of lung metastatic nodules in mice.

Subsequently, experiments were conducted *in vivo* to assess the impacts of circATIC and RCC2. BLca orthotopic implantation model demonstrated that knockdown of circATIC suppressed the fluorescence intensity *in vivo*, whereas forced expression of RCC2 relieved this effect (Fig. 6A and B). The volume of orthotopic implantation tumors also verified this result (Fig. 6C). IHC assay demonstrated that knockdown of circATIC suppressed RCC2 and Ki67 expression level *in vivo*, whereas forced expression of RCC2 relieved this effect (Fig. 6D and E). In addition, Xenograft lung metastasis tumor model showed that knockdown of circATIC suppressed BLca metastasis *in vivo*, whereas forced expression of RCC2 relieved this effect (Fig. 6F and G). The lung histology and morphology were observed with HE staining. HE staining displayed that knockdown of circATIC impaired the number of nodules, meanwhile increased RCC2 expression accelerated the number of nodules (Fig. 6H and I). The findings indicated that increased RCC2 expression reverses the suppressive impact of decreased circATIC levels on cell growth and metastasis *in vitro* and *in vivo*.

### circATIC/LIN28A complex enhance the stability of RCC2 mRNA

**Figure 7:**
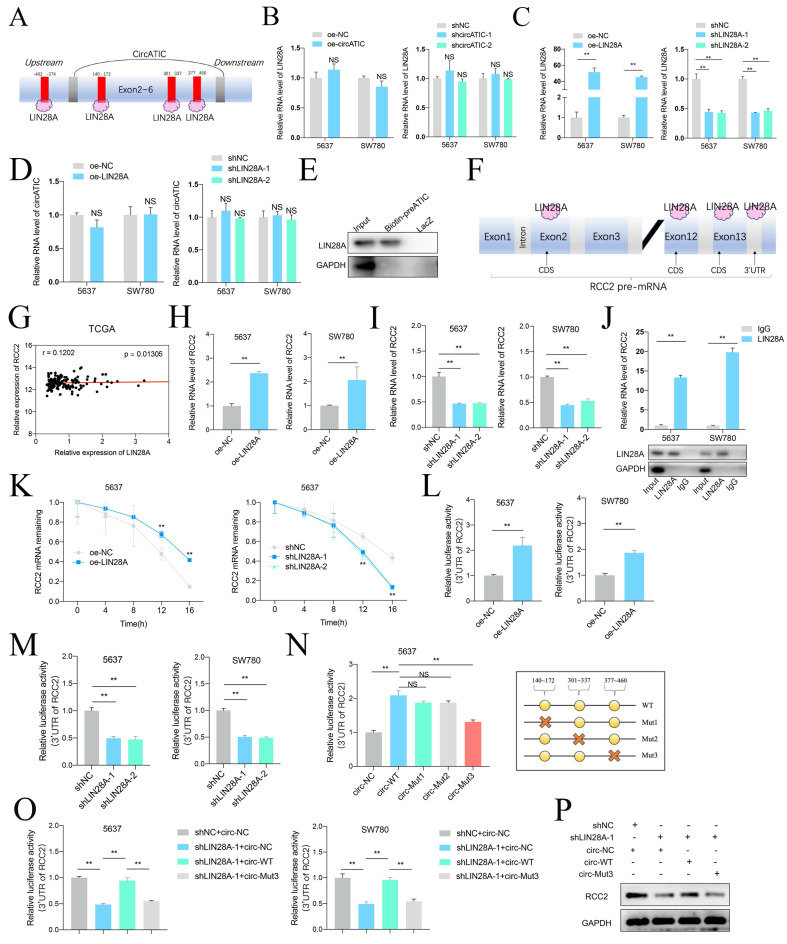
CircATIC/LIN28A complex promoted the stability of RCC2 mRNA. A. Binding sites for LIN28A on circATIC were predicted by CircInteractiome database. B. RT-qPCR showed the expression of LIN28A after transfected with circATIC plasmids in BLca cells. C. The efficiency of LIN28A overexpression or knockdown plasmids in BLca cells. D. LIN28A regulation had no effect on circATIC expression. E. ChIRP assay showed that LIN28A was enriched on pre-circATIC. F. Starbase database predicted the binding site on RCC2 3'UTR for LIN28A. G. The correlation between LIN28A mRNA expression and RCC2 mRNA expression in BLca tissues from TCGA database. H-I. LIN28A positively regulated RCC2 expression in BLca cells. J. RIP assay confirmed that LIN28A binding to RCC2 mRNA. K. RNA stability assay showed that LIN28A promoted the stability of RCC2 mRNA from digestion. L-M. Luciferase activity of RCC2 3'UTR were measured after LIN28A regulation in BLca cells. N. Luciferase activities of RCC2 3'UTR were measured in 5637 cells transfected with control (circ-NC), circATIC (circ-WT), mutant circATIC (circ-Mut1, circ-Mut2 or circ-Mut3). The mutant sites were showed in panel (right). O. Luciferase activities of RCC2 3'UTR were measured in BLca cells transfected with control (shNC), circ-NC, shLIN28A or circ-Mut3. P. Western blot assays showed the expression of RCC2 in BLca cells transfected with control (shNC), circ-NC, shLIN28A or circ-Mut3.

Prior research has shown that circular RNAs can increase the durability of messenger RNAs by interacting with particular proteins. Given that LIN28A is able to maintain RNA stability, we investigate whether circATIC could bind to LIN28A and applied CircInteractome database to forecast the potential binding site (Fig. 7A, Fig. S4A and B). Overexpression or knockdown of circATIC could not influence the expression level of LIN28A (Fig. 7B and C). Moreover, overexpression or knockdown of LIN28A also could not change the circATIC expression level (Fig. 7D). ChIRP assay demonstrated that circATIC directly bound to LIN28A (Fig. 7E). Next, the Starbase database was utilized to examine the connection between LIN28A and the 3'UTR of RCC2 (Fig. 7F, Fig. S4C). In BLca tissues from the TCGA database, there was a positive correlation between the expression of LIN28A mRNA and RCC2 mRNA (Fig. 7G). Overexpression of LIN28A enhanced RCC2 mRNA expression, whereas knockdown of LIN28A caused opposite effect (Fig. 7H and I). RIP assay confirmed that LIN28A directly bound to RCC2 mRNA (Fig. 7J). The RNA stability test indicated that the durability of RCC2 mRNA increased with LIN28A overexpression and decreased with LIN28A suppression (Fig. 7K). Overexpression of LIN28A obviously strengthened the luciferase activity of RCC2 3'-UTR luciferase reporter (Fig. 7L), whereas suppression of LIN28A caused opposite effect (Fig. 7M). Further dual-luciferase reporter assay verified that LIN28A bound to the 377-470 region of circATIC (Fig. 7N). Furthermore, silencing of LIN28A resulted in a reduction in luciferase activity of RCC2 3'UTR luciferase reporter, while upregulation of circATIC reversed this impact and mutation of circATIC was unable to reverse this effect (Fig. 7O). Finally, WB assay demonstrated that knockdown of LIN28A restrained RCC2 protein expression, whereas overexpression of circATIC reversed this effect and circATIC mutation failed to reverse this effect (Fig. 7P). These findings suggest that circATIC strengthens RCC2 mRNA stability via constructing a circATIC/ LIN28A/RCC2 RNA-protein ternary complex.

### circATIC regulated RCC2 expression to promote DDP resistance in BLca cells

**Figure 8:**
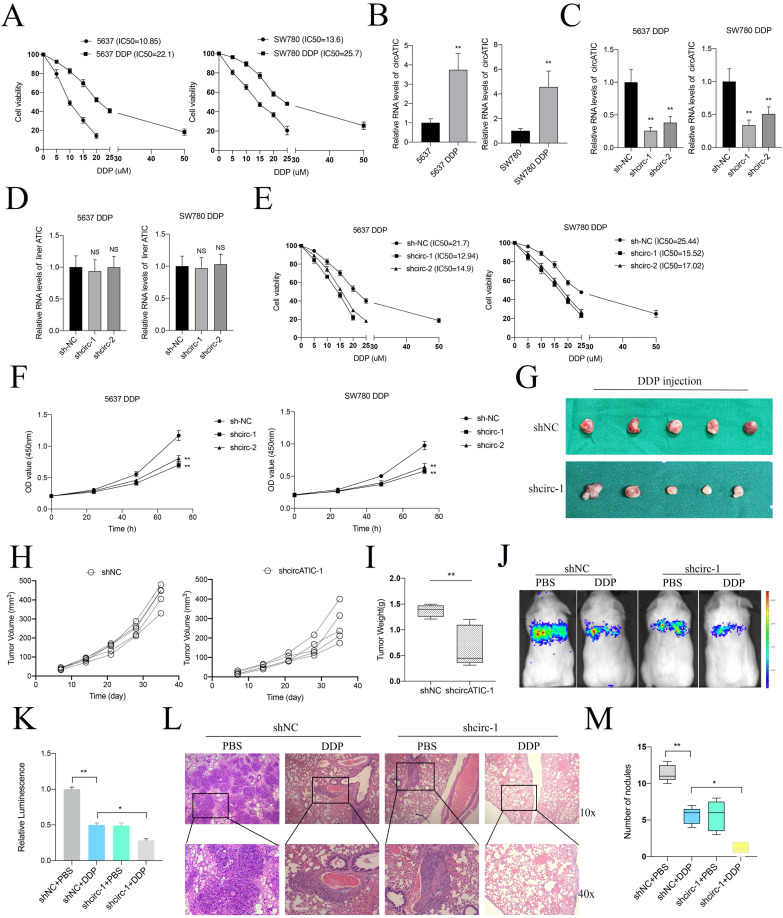
CircATIC promoted DDP resistance of BLca cells *in vitro and in vivo*. A. The DDP-resistant cell lines were constructed and confirmed by cell viability assay. B. The expression of circATIC in cell lines were detected by RT-qPCR. C. The expression of circATIC in DDP-resistant cells estimated by RT-qPCR. D. The expression of ATIC in DDP-resistant cells estimated by RT-qPCR. E. The cell viability in DDP-resistant 5637 cells and DDP-resistant SW780 cells detected by CCK-8 assay. F. The proliferation in DDP-resistant 5637 cells and DDP-resistant SW780 cells detected by CCK-8 assay. G. Xenograft subcutaneous nodules were harvested from mice after DDP treatment. H-I. The volume and weight of tumor in mice upon DDP treatment. J-K. The luminescence of lung metastatic nodules in mice bearing DDP or PBS treatment. L. Comparison of lung metastatic nodules by HE staining. M. The number of lung metastatic nodules in mice.

Previous studies suggested that RCC2 contributed to cisplatin (DDP) resistance in hepatocellular carcinoma and ovarian cancer 30, 31. Hence, we suspected that whether circATIC regulated RCC2 expression to promote DDP resistance in BLca cells and constructed DDP-resistant BLca cell lines (5637 DDP and SW780 DDP) *in vitro* (Fig. 8A). As expected, we discovered that circATIC expression was dramatically increased in 5637 DDP and SW780 DDP cells (Fig. 8B). Knockdown of circATIC in DDP-resistant BLca cell lines could not modify ATIC mRNA expression (Fig.8C and D). Knockdown of circATIC impaired the IC50 value of DDP-resistant BLca cell lines (Fig. 8E). The CCK-8 assay showed that reducing circATIC levels inhibited the growth of DDP-resistant BLca cell (Fig. 8F). *In vivo* experiments demonstrated that the size and mass of transplanted tumors in the circATIC knockdown group treated with DDP were significantly smaller compared to those in the negative control group treated with DDP (Fig. 8G-I). Furthermore, tumor metastasis model showed that decreased circATIC expression effectively restrained BLca metastasis upon DDP treatment (Fig. 8J-K). In Fig. 8L-M, the quantity of nodules in the circATIC knockdown group receiving DDP treatment was significantly lower compared to the negative control group receiving DDP treatment. The findings suggested that circATIC boosted the expression of RCC2 to increase resistance to DDP in BLca cells both *in vitro* and* in vivo*.

### circATIC promoted DDP resistance in BLca cells via accelerating EMT progression and activating JNK pathway

**Figure 9:**
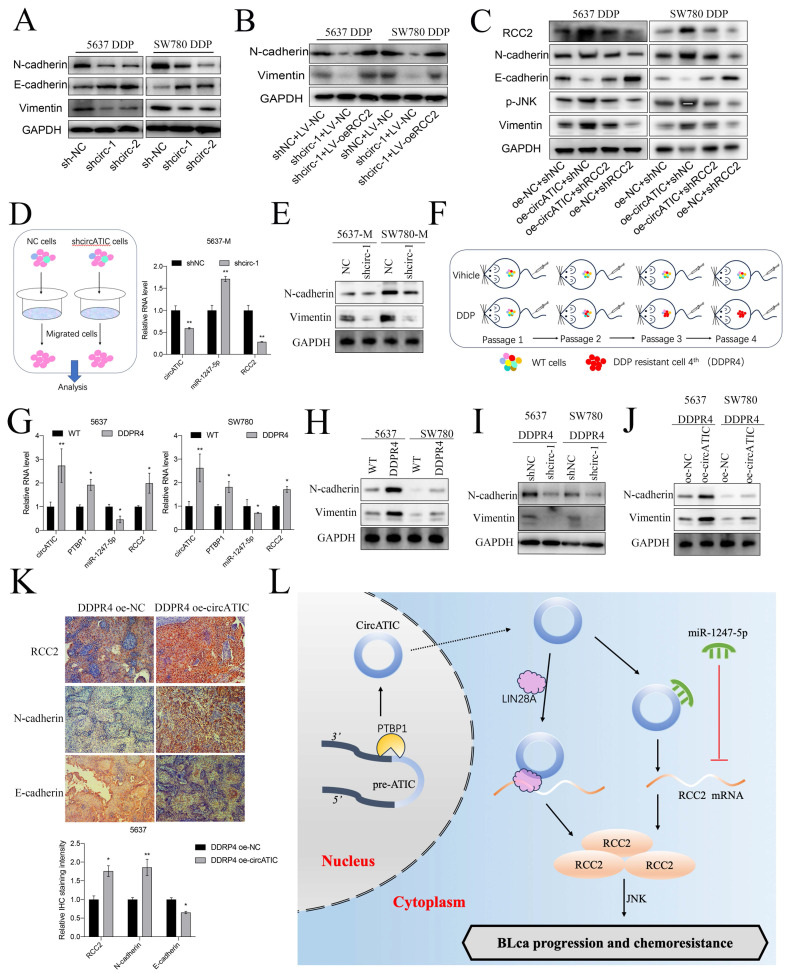
CircATIC enhanced metastasis and DDP resistance of BLca cells via accelerating EMT progression mediated by RCC2/JNK signal pathway. A. Western blotting assay showed the expression of EMT markers in DDP-resistant cells upon circATIC knockdown. B. Western blotting assay showed forced expression of RCC2 rescued the EMT progression suppression induced by circATIC knockdown. C. Western blotting assay showed the protein expression level of RCC2/JNK and EMT markers in DDP-resistant cells transfected with control (shNC or oe-NC), overexpression of circATIC (oe-circATIC) or knockdown of RCC2 (shRCC2). D. Migrated cells (5637-M and SW780-M) were collected from transwell assay (left), RT-qPCR showed the expression of circATIC, miR-1247-5p and RCC2 in migrated cells. E. Western blot experiment showed the protein level of EMT markers in migrated cells. F. Schematic representation of four passages of purified DDP-resistant cells (DDPR4) obtained *in vivo*. G. RT-qPCR showed the expression of PTBP1, circATIC, miR-1247-5p and RCC2 in DDPR4. H-J. The protein expression level of EMT markers in wild type cells (WT) and DDPR4 was measured by western blot assay. K. The IHC staining of EMT markers in DDPR4 nodules. L. Schematic representation of circATIC biogenesis and the mechanism by which circATIC promotes progression and DDP resistance in BLca.

Finally, we preliminarily investigate the molecular mechanisms for DDP resistance associated with circATIC. Previous studies suggested that EMT progression contributed to cisplatin (DDP) resistance in various cancers 32. Interestingly, RCC2 could accelerated the EMT progression in breast cancer, prostate cancer and lung adenocarcinoma 33-35. Besides, RCC2 promoted cancer progression via activating JNK pathway which was closely associated with DDP sensitivity 36, 37. TCGA database predicted that RCC2 expression was significantly correlated with JNK markers in BLca tissues (Fig. S4D and E). The western blot assay showed that reducing circATIC levels increased E-cadherin levels and decreased N-cadherin and vimentin levels by inhibiting RCC2 expression (Fig. 9A). Nevertheless, upregulation of RCC2 counteracted the inhibition of N-cadherin and vimentin expression caused by circATIC depletion (Fig. 9B). Knockdown of PTBP1 suppressed N-cadherin, Vimentin and RCC2 expression and inhibited the activity of JNK pathway (Fig. S5A). Increased levels of circATIC could boost RCC2 levels, leading to the inhibition of E-cadherin expression, the increase of N-cadherin and vimentin expression, and the activation of the JNK pathway, whereas inhibiting RCC2 could counteract these outcomes (Fig. 9C).

Next, we collected the migrated cells transfected with shcircATIC or negative control for further experiments. qRT-PCR assay suggested that circATIC and RCC2 expression was significantly increased in migrated cells, whereas miR-1247-5p expression was decreased (Fig. 9D, Fig. S5B). WB assay demonstrated that N-cadherin and Vimentin protein expression was significantly increased in migrated cells (Fig. 9E). IHC staining with xenograft subcutaneous showed that suppression of circATIC impaired N-cadherin protein expression and strengthened E-cadherin protein expression (Fig. S5C). Finally, xenograft subcutaneous tumor model treated with DDP was applied to construct the fourth generation of DDP-resistant cells (DDPR4) *in vivo* (Fig. 9F). circATIC, PTBP1 and RCC2 mRNA expression was dramatically enhanced in DDPR4, whereas miR-1247-5p expression was dramatically decreased in DDPR4 (Fig. 9G). WB assay demonstrated that N-cadherin and Vimentin protein expression was significantly increased in DDPR4 (Fig. 9H). Silencing of circATIC led to reduced levels of N-cadherin and Vimentin proteins in DDPR4 (Fig. 9I), while upregulation of circATIC had the opposite impact (Fig. 9J). IHC assay presented that RCC2 and N-cadherin were significantly increased in DDPR4, whereas E-cadherin was decreased (Fig. S5D). Overexpression of circATIC enhanced RCC2, N-cadherin protein expression and inhibited E-cadherin protein expression in DDPR4 *in vivo* (Fig. 9K). These results demonstrated that circATIC promoted DDP resistance in BLca cells via accelerating EMT progression and activating JNK pathway (Fig. 9L).

## Discussion

BLca is a frequently occurring cancer in the urinary system that tends to progress quickly and has a poor outcome, posing a significant risk to human health. Despite being a common chemotherapeutic approach for BLca, DDP-based chemotherapy often results in treatment failure and tumor recurrence due to the development of DDP resistance in most patients 38, 39. Previous studies suggested that EMT, DNA damage repair and cancer stem cells (CSCs) contributed to DDP resistance 40-42. In our study, we identified that circATIC was up-regulated in BLca tissues and cells, causing poor prognosis to BLca patients. circATIC was found to enhance growth, metastasis and cisplatin resistance both *in vitro* and *in vivo*. Mechanistically, circATIC sponged miR-1247-5p to enhance RCC2 expression, promoting EMT progression and activating JNK signal pathway. Moreover, circATIC bound with LIN28A to the 3'UTR of RCC2, which enhancing RCC2 mRNA stability. These findings suggest that circAITC may become a potential therapeutic target for chemo-resistant BLca.

With the development of circRNA-sequencing, an ocean of circRNAs were identified in various tissues and organs. It is well known that circRNAs play important roles in cell differentiation, cell development, immune response and organ development 43-46. However, dysregulation of circRNAs cause tumorigenesis, tumor metastasis, chemoresistance and relapse 47, 48. circNT5E promoted BLca growth and metastasis via absorbing miR-502-5p 49. Hsa_circ_0001361 enhanced the metastasis of BLca via miR-491-5p/MMP9 axis 50. CircNDUFB2 inhibited the advancement of lung cancer by breaking down IGF2BPs and stimulating anti-cancer immunity 51. The interaction between circLIFR and MSH2 increases sensitivity to DDP by modulating the MutSα/ATM-p73 pathway in bladder cancer 15. The circ0008399 gene collaborated with WTAP to enhance the formation of the WTAP/METTL3/METTL14 complex, leading to increased resistance to DDP in BLca 14. In addition, previous study showed that various regulatory factors contributed to the biogenesis of circRNAs via binding to their precursor mRNA, such as QKI, EIF4A3, FUS and PTBP1 52-55. PTBP1 is an RNA-binding protein, which belongs to the family of heterogeneous nuclear ribonucleoprotein (hnRNP), getting involved in mRNA stability, mRNA translation, and localization 26. Among these, PTBP1 acts as a splicing factor to regulate alternative splicing of precursor mRNA 56. In glioma, PTBP1 bound to the pre-mRNA of ANKRD17 to promote circANKRD17 expression 57. In colorectal cancer, PTBP1 synergistically enhances the formation of circTDRD3 under hypoxic conditions 55. Numerous studies demonstrated that PTBP1 promoted the progression of various cancers, such as glioma, colorectal cancer, breast cancer, BLca and gastric cancer 26, 58. In BLca, PTBP1 expression was dramatically upregulated in BLca tissues, causing poor prognosis to BLca patients. Experiments conducted *in vitro and in vivo* showed that PTBP1 accelerated the growth and metastasis of cancer cells 59. Additionally, PTBP1 may increase the repellence of cisplatin in certain types of cancer. In osteosarcoma, PTBP1 enhance the sensitivity of cisplatin in osteosarcoma cell lines via accelerating the degradation of SLC31A1 mRNA 60. In hepatocellular carcinoma, PTBP1 could promote cisplatin resistance via strengthening glutamine uptake and enhancing glutaminase (GLS) expression 61. In our study, we applied the CircInteractome database to predicted the splicing factors interacted with circATIC and discovered that PTBP1 bound with the pre-mRNA of ATIC to promote circATIC biogenesis.

To explore the mechanism of circATIC promoting cancer, it is necessary to clarify its subcellular localization. Nuclear circRNAs are normally involved in transcriptional regulation 62, while cytoplasmic circRNAs can adsorb miRNAs or proteins, or even translate new functional proteins, thus participating in post-transcriptional modification and post-translational regulation 63-65. The study revealed that circATIC was primarily localized in the cytoplasm, indicating its role as a 'miRNA sponge' or 'protein sponge'. Expression analysis, TRAP assay and dual luciferase reporter assay were used to verify that miR-1247-5p was the target gene of circATIC. MiR-1247-5p plays a tumor suppressor role in multiple cancers 27, 29, 66. Our studies validated the significant decrease of miR-1247-5p in 52 BLca tissues compared to normal tissues, leading to enhanced proliferation, migration and invasion of BLca cells via targeting Regulator of chromosome condensation 2 (RCC2). In addition, we discovered that circATIC strengthens RCC2 mRNA stability via constructing a circATIC/LIN28A/RCC2 RNA-protein ternary complex. A large number of studies have proved that circRNAs serve as modulators to influence the stability of mRNAs. The presence of CircSTX6 may increase the stability of SUZ12 mRNA by creating a circSTX6/PABPC1/SUZ12 RNA-protein complex, leading to the advancement of BLca and resistance to cisplatin 18. CircXPO1 bound with IGF2BP1 to enhance CTNNB1 mRNA stability, promoting the progression of lung adenocarcinoma 67. Within liver cancer, circNSUN2 interacted with IGF2BP2 to create a circNSUN2/IGF2BP2/HMGA2 RNA-protein complex in the cytoplasm, ultimately boosting the durability of HMGA2 mRNA 68. In our study, circATIC could enhance RCC2 mRNA stability via sponging miR-1247-5p or constructing a circATIC/ LIN28A/RCC2 RNA-protein ternary complex.

RCC2 is a member of RCC1 family and regarded as a telophase disk-binding protein in the course of mitosis, accelerating the progression of prometaphase to metaphase 69. Multiple studies have shown that RCC2 functions as an oncogene in various cancer, enhancing cell growth, metastasis and resistance to drugs 70. RCC2 in breast cancer triggers the Wnt signaling pathway, leading to the acceleration of EMT and development of cancer cells 33. In lung adenocarcinoma, RCC2 promote cell metastasis via activating MAPK-JNK signal pathway to enhance EMT progression 35. In prostate cancer, RCC2 enhances cell proliferation and migration via modulating Hh/GLI1 signaling pathway 34. In lung cancer and ovarian cancer, RCC2 suppresses cell apoptosis and enhances the sensitivity of chemotherapy drugs via activating Rac1/JNK pathway 36. In hepatocellular carcinoma, RCC2 facilitates cell metastasis and DDP resistance via activating AKT signal pathway 30. As we known, EMT progression and JNK signal pathway are closely correlated with DDP resistance. In breast cancer, EMT progression contribute to DDP resistance and radio-resistance 71. Cholangiocarcinoma cells show increased resistance to DDP through the activation of EMT progression by the PLCB1-PI3K-AKT signaling pathway 72. In lung cancer, suppression of ATM enhanced the sensitivity of DDP and EMT progression via JAK/STAT3/PD-L1 pathway 73. In BLca, ADNP enhanced DDP resistance via regulating TGF-β-mediated EMT pathway 74. Our research revealed that circATIC enhances RCC2 expression, leading to increased EMT progression and ultimately contributing to enhanced DDP resistance both *in vitro* and *in vivo*. Besides, RCC2 could activate JNK signal pathway to enhance DDP resistance. Belonging to the MAPK family, JNK plays a role in advancing cancer and developing resistance to drugs like cisplatin and gemcitabine. The JNK pathway is crucial in the development of resistance to DDP in ovarian cancer 75. In colon cancer, suppression of JNK signal pathway enhanced the sensitivity to various drugs, including oxaliplatin, SN-38, and 5-FU. In bladder cancer, PDCD4 modulate DDP sensitivity via regulating JNK/c-Jun signaling pathway 76. Besides, MLN4924 promotes DDP-induced cytotoxicity via regulating JNK signal pathway 77. In our study, we demonstrated that circATIC promoted RCC2 expression to enhance EMT progression and activate JNK signal pathway, thus strengthening DDP resistance in BLca cells.

In conclusion, our results demonstrate that circATIC may act as a prognostic indicator in BLca and targeting on circATIC is a potential method for the treatment in DDP resistance BLca.

## Supplementary Material

Supplementary figures and tables.

## Figures and Tables

**Table 1:** Correlation between circATIC expression level and clinical characteristics in 52 Urothelial carcinoma of the bladder (UCB) patients.

Characteristics		Total	circATIC expression		P value
			High	Low	
Gender	Male	33	26	7	0.674
	Female	19	14	5	
Age (years)	< 60	23	19	4	0.386
	≥ 60	29	21	8	
Tumor size (cm)	< 3cm	22	11	11	0.000^*^
	≥ 3cm	30	29	1	
Histological grade	Low	21	18	3	0.216
	High	31	22	9	
T stage	T_1_/T_2_	29	23	6	0.646
	T_3_/T_4_	23	17	6	
Lymphatic metastasis	Absent	49	37	12	0.328
	Present	3	3	0	
Postoperative metastasis	Metastasis	24	22	2	0.019*
	Non-metastasis	28	18	10	

*P < 0.05 represents statistical significance. Results are analyzed by Chi-square test.
