# Supplementary material for: PTBP1-mediated biogenesis of circATIC promotes progression and cisplatin resistance of bladder cancer
Source: Int J Biol Sci. 2024 Jun 24;20(9):3570–89. doi: 10.7150/ijbs.96671 (PMC11234215; doi:10.7150/ijbs.96671)
Supplement: Supplementary file 1 — Supplementary figures and tables. [file ijbsv20p3570s1.pdf]

A

circRNA	Tag Name	% Identity	Alignment Length	Mismatches	Gap Openings	Tag Start	Tag End	circRNA Start	circRNA ENd	Upstream/Downstream
hsa_circ_0058058	HHFPT_162394_PTB_cluster-13357_8_18_47	100.00	47	0	0	1	47	0	+512	Downstream
hsa_circ_0058058	HHFPT_162395_PTB_cluster-13357_9_20_85	100.00	85	0	0	1	85	+734	+818	Downstream

B

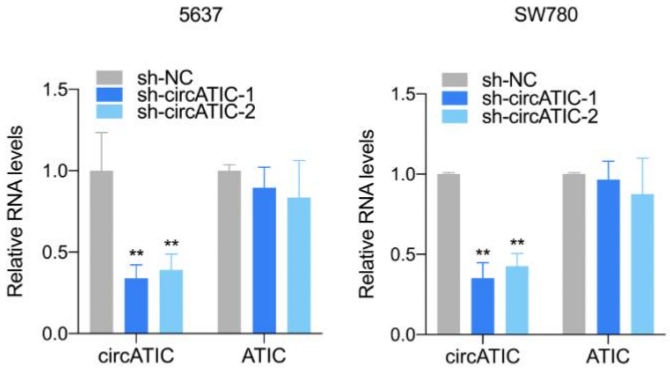

Figure S1. PTBP1-induced circATIC inhibited tumor growth. A. The binding sites between PTBP1 and circATIC predicted by CircInteractome database. B. RT-qPCR confirmed the knockdown of circATIC by shRNAs.

A

circRNA	Tag Name	% Identity	Alignment Length	Mismatches	Gap Openings	Tag Start	Tag End	circRNA Start	circRNA ENd	Upstream/Downstream
hsa_circ_0058058	HHFCT_92387_cluster-13357_1_11_44	100.00	44	0	0	1	44	-380	-337	Upstream
hsa_circ_0058058	HHFCT_92392_cluster-13357_7_16_48	100.00	48	0	0	1	48	0	+516	Downstream
hsa_circ_0058058	HHFCT_92393_cluster-13357_8_8_27	100.00	27	0	0	1	27	+664	+690	Downstream
hsa_circ_0058058	HHFCT_92394_cluster-13357_9_22_41	100.00	41	0	0	1	41	+734	+774	Downstream
hsa_circ_0058058	HHFCT_92395_cluster-13357_10_9_44	100.00	44	0	0	1	44	+789	+832	Downstream
hsa_circ_0058058	HHFKP_85667_cluster-13357_5_18_41	100.00	41	0	0	1	41	+721	+761	Downstream
hsa_circ_0058058	HHRBL_8808_BCBL1-2of3-SCL_3157_93	100.00	93	0	0	1	93	+710	+802	Downstream

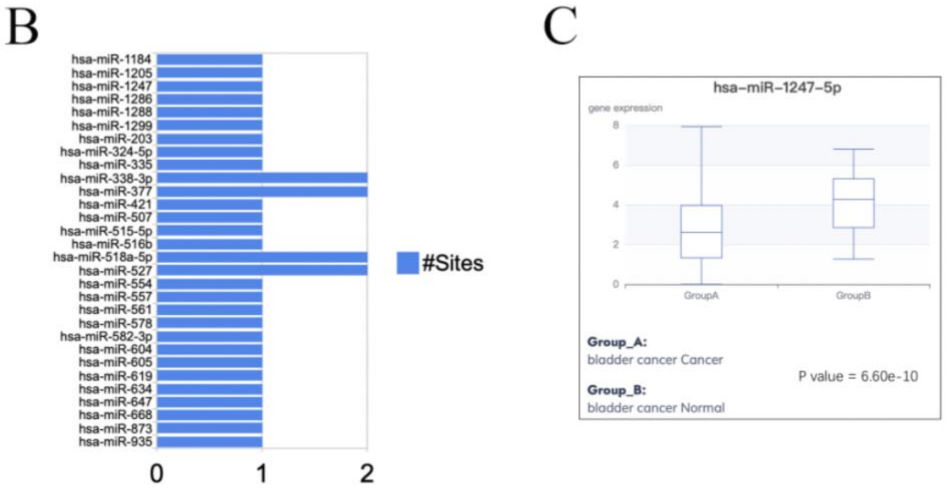

D

CircRNA Mirbase ID	CircRNA (Top) - miRNA (Bottom) pairing	Site Type	CircRNA Start	CircRNA End
hsa_circ_0058058 (5' ... 3')<br>hsa-miR-1247 (3' ... 5')	AGAUGUCUCUGAGUUGACGGGAU<br>|||||| AGGCCCCUGCUUGCCCUGCCCA	7mer-1a	143	149

Figure S2. CircATIC sponges miR-1247-5p. A. The binding sites between AGO2 and circATIC predicted by CircInteractome database. B. miRNAs predicted to bind with circATIC. C. miR-1247-5p expression in bladder cancer tissues compared to normal tissues according to TCGA database. D. The core binding sequence between circATIC and miR-1247-5p.

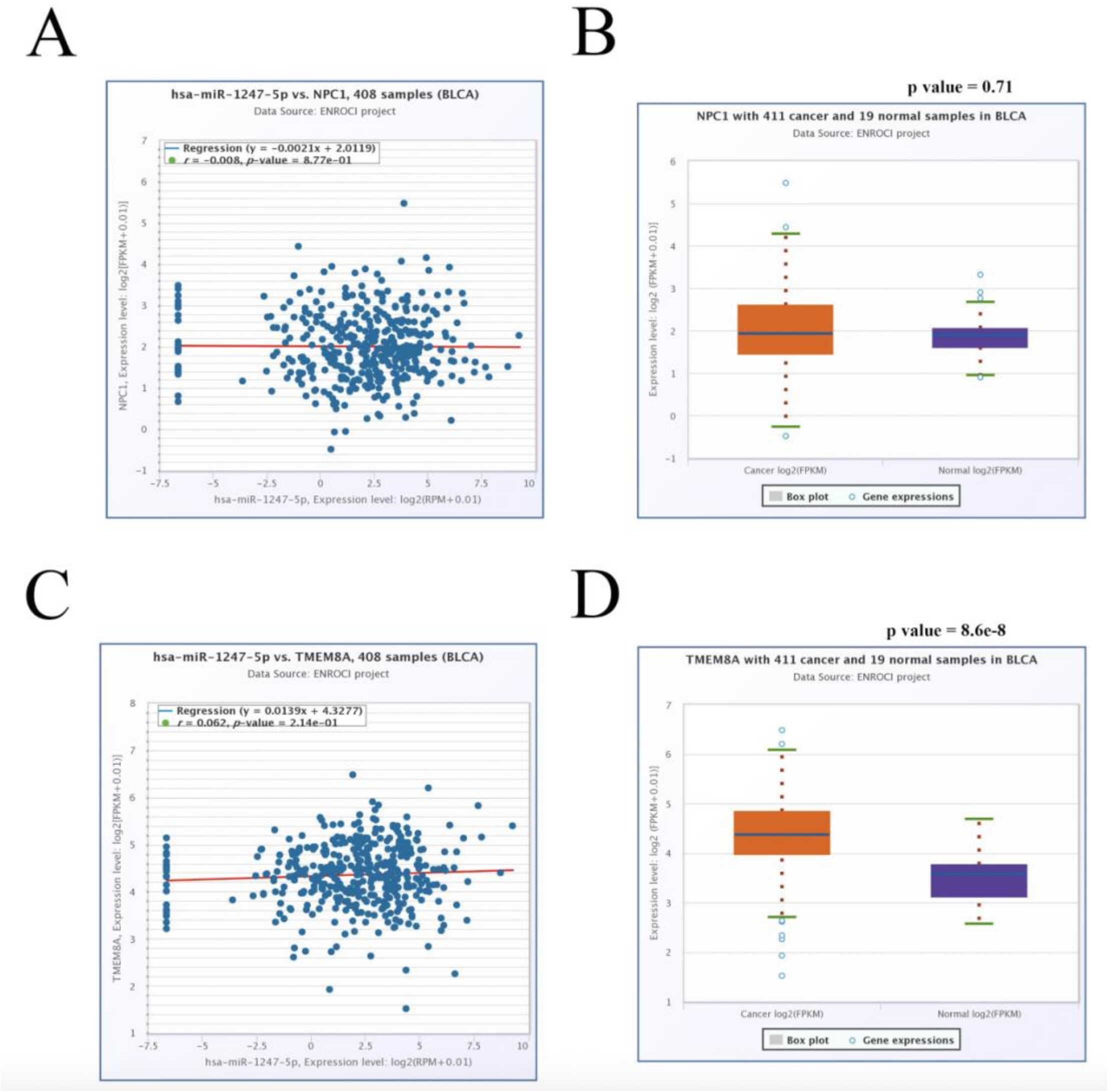

Figure S3. Bioinformatics analysis predicted target genes of miR-1247-5p. A. Starbase database showed that NPC1 expression was negatively correlated with miR-1247-5p expression in bladder cancer tissues. B. Starbase database showed that NPC1 was not dysregulated in bladder cancer tissues. C. Starbase database showed that TMEM8A expression was positively correlated with miR-1247-5p expression in bladder cancer tissues. D. Starbase database showed that TMEM8A was up-regulated in bladder cancer tissues (n=411) comparing to normal tissues (n=19).

A

circRNA	Tag Name	% Identity	Alignment Length	Mismatches	Gap Openings	Tag Start	Tag End	circRNA Start	circRNA ENd	Upstream/Downstream
hsa_circ_0058058	HPTLA_21972_LIN28A_G17174_5_ENST0000443953_ATIC_5utr_29	100.00	29	0	0	1	29	-402	-374	Upstream

B

Circular RNA	Tag Name	% Identity	Alignment Length	Mismatches	Gap Openings	Tag Start	Tag End	Circular RNA Start	Circular RNA ENd
hsa_circ_0058058	HHYLH_3621_LIN28_H9_15045_33	100.00	33	0	0	1	33	140	172
hsa_circ_0058058	HHYLV_1997_LIN28_V5_15045_37	100.00	37	0	0	1	37	301	337
hsa_circ_0058058	HHYLV_1998_LIN28_V5_15045_84	100.00	84	0	0	1	84	377	460

C

Transcript ID	Transcript Name	Binding Exon	Binding Region
ENST00000375436	RCC2-202	Exon-13	3'UTR
ENST00000474892	RCC2-203	Exon-2	Exon

Transcript ID	Transcript Name	Binding Exon	Binding Region
ENST00000375436	RCC2-202	Exon-13	CDS
ENST00000375433	RCC2-201	Exon-12	CDS

D

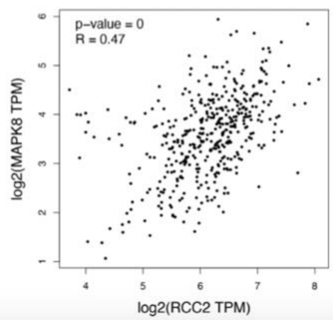

E

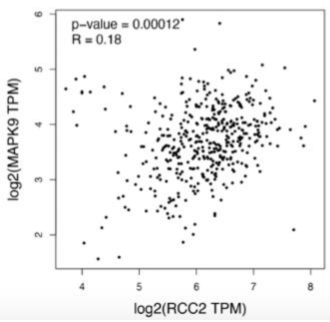

Figure S4. circATIC/LIN28A complex binds to RCC2 and activate JNK. A. The binding site between LIN28A and ATIC pre-mRNA predicted by CircInteractome database. B. The binding site between LIN28A and circATIC predicted by CircInteractome database. C. The binding site between LIN28A and RCC2 transcript predicted by Starbase database. D-E. TCGA database predicted that RCC2 expression was significantly correlated with MAPK9 and MAPK8.

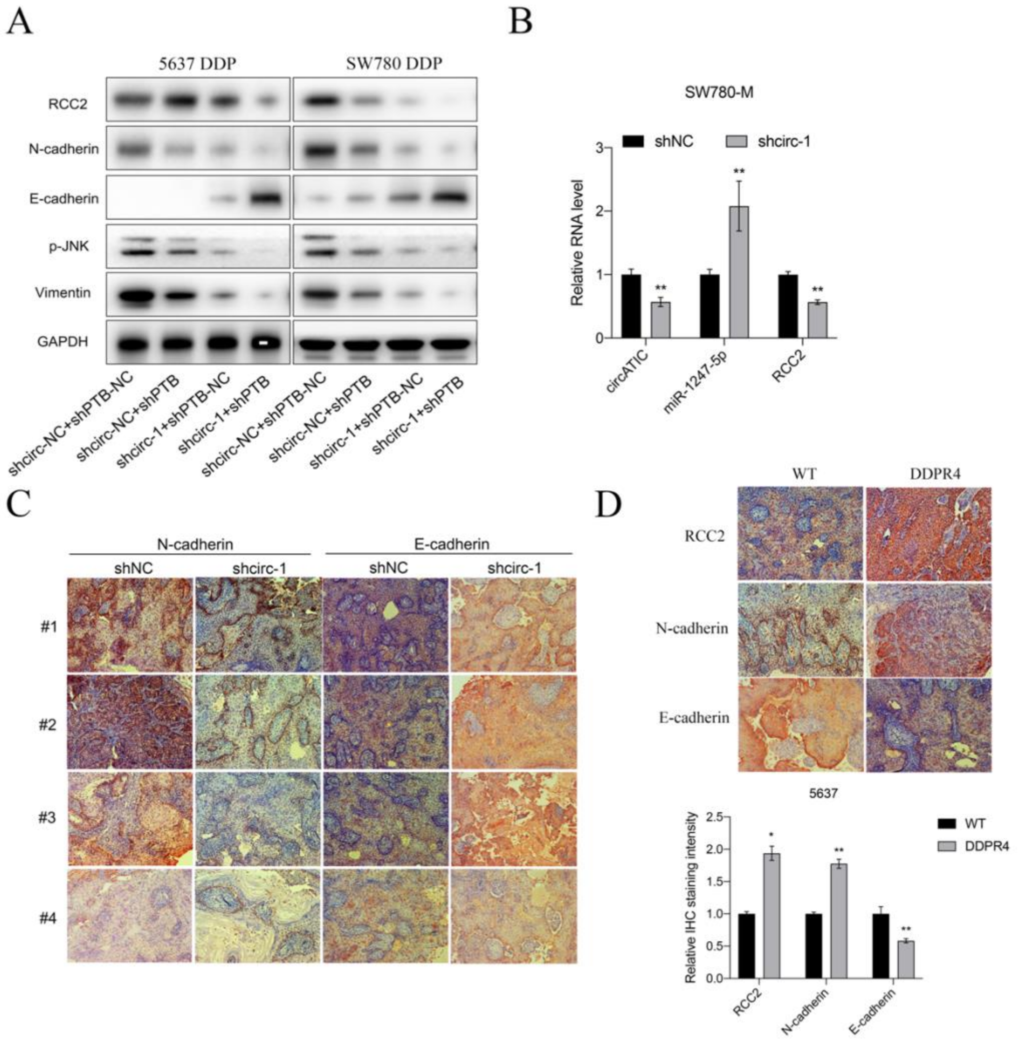

Figure S5. CircATIC promoted EMT and activated RCC2/JNK in DDP-resistant cells. A. EMT markers expression and RCC2/JNK expression in DDP-resistant cells. B. RT-qPCR showed the expression of circATIC, miR-1247-5p and RCC2 in migrated cells. C. The IHC staining of EMT markers in xenograft subcutaneous nodules. D. The IHC staining of RCC2 and EMT markers in DDPR4 nodules.

Supplementary Table S1. List of primers

Gene	Sequence (5'-3')	
circATIC	F: ACGCCAGTTAGCCTTGAAGC	R: CCTCCGGAAGCGACCAGATT
ATIC	F: CGGCTCTTCCACCACTGATT	R: GGCGTGACTGTTCACCTACA
GAPDH	F: CAAGGCTGAGAACGGGAAG	R: TGAAGACGCCAGTGGACTC
U6	F: CGCTTCGGCAGCACATATAC	R: TTCACGAATTTGCGTGTCAT
miR-1247-5p	F: CCCGTCCCGTTCGT	R: GTCCAGTTTTTTTTTTTTTTTCCG
miR-203a	F: CAGGTGAAATGTTTAGGACCA	R: GGTCCAGTTTTTTTTTTTTTTTCTAGT
miR-335	F: GCAGTCAAGAGCAATAACGA	R: CAGGTCCAGTTTTTTTTTTTTTTTACA
miR-338-3p	F: GCAGTCCAGCATCAGTG	R: GGTCCAGTTTTTTTTTTTTTTTCAAC
miR-377	F: GCAGATCACACAAAGGCA	R: GTCCAGTTTTTTTTTTTTTTTACAAAAGT
miR-515-5p	F: CAGTTCTCCAAAAGAAAGCAC	R: GTCCAGTTTTTTTTTTTTTTTCAGAAAG
miR-605-3p	F: GCAGAGAAGGCACTATGAG	R: GGTCCAGTTTTTTTTTTTTTTTCTAAATC
miR-125a-5p	F: CCCTGAGACCCTTTAACCT	R: GGTCCAGTTTTTTTTTTTTTTTCACA
miR-1301-3p	F: GCTGCCTGGGAGTGA	R: GGTCCAGTTTTTTTTTTTTTTTGAAG
miR-1205	F: AGTCTGCAGGGTTTGCT	R: GGTCCAGTTTTTTTTTTTTTTTCTCA
miR-1299	F: CAGTTCTGGAATTCTGTGTGA	R: GTCCAGTTTTTTTTTTTTTTTCCCT
miR-557	F: CGGGTGGGCCTTG	R: GGTCCAGTTTTTTTTTTTTTTTAGAC
miR-619-3p	F: ACCTGGACATGTTTGTGC	R: CAGGTCCAGTTTTTTTTTTTTTTTACT
miR-619-5p	F: GCTGGGATTACAGGCATGA	R: TCCAGTTTTTTTTTTTTTTTGGCT
RCC2	F: GCGCACTCGCCAATTAAGAG	R: GCACAGAAAGGGCTGATCCT
PTBP1	F: AGCGCGTGAAGATCCTGTTC	R: CAGGGGTGAGTTGCCGTAG
LIN28A	F： CTTTTGCCAAAGCATCAACC3	R： GGGCTGTGGATCTCTTCCTC

Supplementary Table S2. Sequences of plasmids

shRNA	Sequence (5’ -3’)
shcirc-NC（shNC）	GTTCTCCGAACGTGTCACGT
circATIC shRNA-1(shcirc-1)	GTTAGCCTTGAAGCCTTATTT
circATIC shRNA-2(shcirc-2)	GCCTTGAAGCCTTATTTAGTG
shNC	CAACAAGATGAAGAGCACCAA
shPTBP1	GCACAGAAAGGGCTGATCCT
circATIC WT	ATCTGGTCGCTTCCGGAGGGACTGCAAAAGCTCTCA GGGATGCTGGTCTGGCAGTCAGAGATGTCTCTGAGTT GACGGGATTTCCTGAAATGTTGGGGGGACGTGTGAA AACTTTGCATCCTGCAGTCCATGCTGGAATCCTAGCT CGTAATATTCCAGAAGATAATGCTGACATGGCCAGAC TTGATTTCAATCTTATAAGAGTTG
circATIC Mut	ATCTGGTCGCTTCCGGAGGGACTGCAAAAGCTCTCA GGGATGCTGGTCTGGCAGTCAGAGATGTCTCTGAGTT CTGCCCATTTCCTGAAATGTTGGGGGGACGTGTGAA AACTTTGCATCCTGCAGTCCATGCTGGAATCCTAGCT CGTAATATTCCAGAAGATAATGCTGACATGGCCAGAC TTGATTTCAATCTTATAAGAGTTG
RCC2 WT	TGCTCCCGGAGACTCCTCCGACTCCACACCTCTCGCG GCAGCTGTCATTTCCATGTGCACTGGGACGGGAAGT CAAACGAGGAATTTAAAAAAGCAAAAGTTGACCGA AGGTGCATTTTTGTTTAGACTCCCTGAGGT
RCC2 Mut	TGCTCCCGGAGACTCCTCCGACTCCACACCTCTCGCG GCAGCTGTCATTTCCATGTGCACTGCCTGCCCAAGTC AAACGAGGAATTTAAAAAAGCAAAAGTTGACCGAA GGTGCATTTTTGTTTAGACTCCCTGAGGT
oe-RCC2	NM_018715.4
miR-NC mimic	mircON mimic NC #22 (RiboBio, China)
miR-1247-5p mimic	mircON has-miR-1247-5p mimic (RiboBio, China)
5’Biotin ATIC pre-mRNA	GAAACAGTACAGCAAAGGCGTATCTCAGATGCCCTT GAGATATGGAATGAACCCACATCAGACCCCTGCCCA

	GCTGTACACACTG
shRCC2	CAACTCAGATGGGAAGTTCAT
shLIN28A-1	CCTGGTGGAGTATTCTGTATT
shLIN28A-2	GCACCAGAGTAAGCTGCACAT
Circ-NC/circ-WT/circ-Mut1/circ-Mut2/circ-Mut3	Synthesized by RiboBio

Supplementary Table S3. List of antibodies

Antibodies	Source（Identifier）	Application
E-cadherin	Proteintech (20874-1-AP)	Western blot and IHC
N-cadherin	Proteintech (22018-1-AP)	Western blot and IHC
Vimentin	Cell Signaling Technology (#5741)	Western blot
GST	Cell Signaling Technology (#2622)	TRAP
AGO2	Cell Signaling Technology (#2897)	TRAP
RCC2	Proteintech (16755-1-AP)	Western blot and IHC
p-JNK	ZenBio (#381100)	Western blot
GAPDH	Cell Signaling Technology (#5174)	Western blot and TRAP
Ki67	Proteintech (27309-1-AP)	IHC
PTBP1	Proteintech (12582-1-AP)	Western blot and ChIRP
LIN28A	Proteintech (11724-1-AP)	RIP
